# Uncover diagnostic immunity/hypoxia/ferroptosis/epithelial mesenchymal transformation-related CCR5, CD86, CD8A, ITGAM, and PTPRC in kidney transplantation patients with allograft rejection

**DOI:** 10.1080/0886022X.2022.2141648

**Published:** 2022-11-04

**Authors:** Long He, Boqian Wang, Xueyi Wang, Yuewen Liu, Xing Song, Yijian Zhang, Xin Li, Hongwei Yang

**Affiliations:** Organ Transplantation Center, General Hospital of Northern Theater Command, Shenyang City, China

**Keywords:** Kidney transplantation, allograft rejection, immunity, hypoxia, ferroptosis, epithelial mesenchymal transformation

## Abstract

The aim of this study was to identify predictive immunity/hypoxia/ferroptosis/epithelial mesenchymal transformation (EMT)-related biomarkers, pathways and new drugs in allograft rejection in kidney transplant patients. First, gene expression data were downloaded followed by identification of differentially expressed genes (DEGs), weighted gene co-expression network analysis (WGCNA) and protein–protein interaction (PPI) analysis. Second, diagnostic model was construction based on key genes, followed by correlation analysis between immune/hypoxia/ferroptosis/EMT and key diagnostic genes. Finally, drug prediction of diagnostic key genes was carried out. Five diagnostic genes were further identified, including CCR5, CD86, CD8A, ITGAM, and PTPRC, which were positively correlated with allograft rejection after the kidney transplant. Highly infiltrated immune cells, highly expression of hypoxia-related genes and activated status of EMT were significantly positively correlated with five diagnostic genes. Interestingly, suppressors of ferroptosis (SOFs) and drivers of ferroptosis (DOFs) showed a complex regulatory relationship between ferroptosis and five diagnostic genes. CD86, CCR5, and ITGAM were respectively drug target of ABATACEPT, MARAVIROC, and CLARITHROMYCIN. PTPRC was drug target of both PREDNISONE and EPOETIN BETA. In conclusion, the study could be useful in understanding changes in the microenvironment within transplantation, which may promote or sustain the development of allograft rejection after kidney transplantation.

## Introduction

In recent years, kidney transplantation has been considered as the best therapeutic intervention for patients with end-stage organ failure [1]. However, kidney transplantation brings the risk of allograft rejection. If leaving unchecked, allograft rejection reaction can destroy the graft. With the use of immunosuppressive agents, the incidence of transplant rejection has reduced [2]. Although the annual survival rate of kidney transplant has reached more than 90%, there is a 4–5% loss of function of the kidney graft. The 5-year survival rate of kidney transplant is 70%, whereas the 10-year survival rate is only 50% [2]. Regular monitoring of serum creatinine is an insensitive predictor and only increases upon the deficiency in kidney function [3]. Thus, it is important to identify potential diagnostic and therapeutic markers that associated with different molecular mechanisms in the process of allograft rejection in kidney transplant patients.

Activation of the immune system in recipients is majorly responsible for allograft rejection [4,5]. The severity of the allograft dysfunction process is positively correlated with the incidence of T cell-mediated acute rejection [6]. Hypoxia, an inevitable event accompanying kidney transplantation, is regarded as a common cause for delayed graft function [7–10]. In response to hypoxia, tubular epithelial cells can produce multiple pro-inflammatory factors and trigger tubule interstitial inflammation [11–13]. Ferroptosis, characterized by membrane damage, is an iron-dependent and regulated cell death [14]. Ferroptosis-related indicators, including iron and lipid peroxides are associated with renal fibrosis [15–20]. Epithelial–mesenchymal transition (EMT) is the indispensable process in embryonic development and organ fibrosis [21]. It is noted that the EMT is involved in the progression of interstitial fibrosis in kidney allograft with chronic kidney allograft dysfunction [22]. Maybe, there are complex regulatory mechanisms among immunity, hypoxia, ferroptosis, and EMT, which may be important factors in allograft rejection after the kidney transplant. In view of this, the aim of the present study is to explore predictive immunity/hypoxia/ferroptosis/EMT biomarkers, pathways, and new drugs in the process of graft rejection in kidney transplant patients, thus enabling more accurate and less invasive diagnosis.

## Materials and methods

### Filtering of dataset

Gene expression data were downloaded from the Gene Expression Omnibus (GEO) dataset. Keywords of ‘kidney transplant’ and ‘Homo sapiens’ were used to filter the gene expression profile data. The corresponding data set was then filtered using the following criteria. Inclusion criteria for dataset are as follows: (1) there are more than five cases; (2) there is rejection information. Exclusion criteria for dataset are as follows: (1) the study is conducted at the cell line or animal level; (2) there is a single case in the study; (3) repetitive or overlapping study. Finally, a total of four datasets (involving kidney transplant biopsy sample) were included in the study, including GSE36059 (involving 122 patients with allograft rejection and 281 patients without allograft rejection), GSE48581 (involving 78 patients with allograft rejection and 222 patients without allograft rejection), GSE129166 (involving 35 patients with allograft rejection and 60 patients without allograft rejection), and GSE124203 (involving 774 patients with allograft rejection and 905 patients without allograft rejection). Randomly, GSE36059, GSE48581, and GSE129166 datasets were considered as a training set. GSE124203 datasets were considered as a validation set. For the above four datasets, the gene expression matrix files were downloaded and annotated using annotation files of GPL platform. For datasets of GSE36059, GSE48581, and GSE129166, the combat function in ‘SVA’ in R package was utilized to remove batch effect. The combined dataset included 235 cases and 563 normal controls.

### Screening of differentially expressed genes (DEGs) and weighted gene co-expression network analysis (WGCNA)

In the training set, the ‘llimma’ package was used to identify DEGs in kidney transplant patients with allograft rejection. The screening criteria of DEGs were false discovery rate (FDR) <0.05 and |log_2_ fold change (FC)| >0.5. The volcano map was used for visualization of DEGs. The ‘WGCNA’ in R package was utilized to analyze the co-expression network of all genes, followed by the construction of the scale-free gene co-expression network. Genes with similar expression patterns were gathered together. Modular signature genes (ME) were defined as the first major component in each module. To identify the key modules most associated with allograft rejection, the ME of each module was calculated using the ‘moduleEigengenes’ function. Pearson’s method was applied to analyze the correlation with allograft rejection. Modules with the highest positive and negative correlation with allograft rejection were chosen as hub modules.

### Functional analysis and protein–protein interaction (PPI) network construction of common genes in hub modules and DEGs

First, in order to study the function of common genes in hub modules and DEGs in kidney transplant patients with allograft rejection, David database was used for Gene Ontology (GO) analysis. In addition, GSVA analysis was carried out to reveal differences in metabolic pathways. Significantly enriched GO terms and pathways were identified under the threshold value of FDR <0.05. Second, common genes in hub modules and DEGs were put into the STRING database to study the regulatory relationship between proteins encoded by these genes. The PPI network was constructed by Cytoscape software. CytoHubba is one of the plug-ins in Cytoscape software, which provides 11 topology analysis methods [23]. Finally, a total of seven topology analysis methods were adopted to screen central genes, including Degree, EPC, MNC, MCC, Closeness, Betweenness, and Stress. The first 20 node genes of each algorithm score were identified through the R package ‘UpSet’ to screen the multi-center intersection genes, which were considered as key genes involved in allograft rejection after the kidney transplant.

### Construction of diagnostic model based on key genes

The receiver operating characteristic (ROC) curve was used to determine the accuracy of key genes in the diagnosis of allograft rejection after the kidney transplant. The area under curve (AUC) is an evaluation index of model performance. The AUC value ranges from 0 to 1, where 0.7 is acceptable performance and 0.9 is excellent performance. First, ROC curves of the combinations of key genes were plotted. Then, ROC curve of the single key gene was plotted separately in allograft rejection and non-rejection groups. Finally, the accuracy of the model was verified in the validation set.

### Construction of regulatory networks between miRNAs, transcription factors (TFs) and key genes

First, to explore the influence of miRNA-gene regulatory relationship on the occurrence and development of allograft rejection after the kidney transplant, the miRNA-key gene regulatory network was constructed based on the interaction data of miRDB Database. Second, TRRUST Database was used to study the role of TFs in key gene regulation.

### Correlation analysis between immune, hypoxia, ferroptosis, epithelial–mesenchymal transition, and key genes

First, the single-sample gene set enrichment analysis (ssGSEA) algorithm was used to quantify the abundance of each cell infiltrate in the immune microenvironment (IME). Gene sets that mark each infiltrating immune cell type in IME were obtained from previous studies [24,25]. To observe the immune status of kidney transplant patients with allograft rejection, enrichment score was used to represent the relative abundance of each infiltrating cell in IME in each sample. Second, the status of hypoxia in the kidney transplant patients with allograft rejection was inferred from the hypoxia marker gene set in the MSigDB Database, which includes 200 hypoxia-related genes. Third, status of ferroptosis and EMT in the kidney transplant patients with allograft rejection was inferred from the literature [26,27]. Finally, the correlation between immune, hypoxia, ferroptosis, EMT and key genes was analyzed.

### Drug prediction of key genes

In order to provide a new perspective for disease diagnosis, treatment, and research for kidney transplant patients with allograft rejection, drugs related to key genes were screened out based on DGIdb Database (https://dgidb.org/).

### Statistical analysis

Statistical analysis was performed using R version 3.5.3 (R Foundation for Statistical Computing, Vienna, Austria). The Limma package was used for differential expression analysis. Modules positively associated with allograft rejection were screened using the ‘WGCNA’ package. The function of the gene set was studied by using David database. The regulation relationship between the gene set was performed by using STRING database. ROC analysis was performed using the R package ‘pROC’ to calculate the AUC to assess the accuracy of genes in the diagnosis of allograft rejection. Wilcoxn.test was used to compare the differences of different immune cells in allograft rejection. Pearson’s correlation analysis was used to analyze the relationship between genes and immune cells.

## Results

### Identification of DEGs

**Figure 1.**
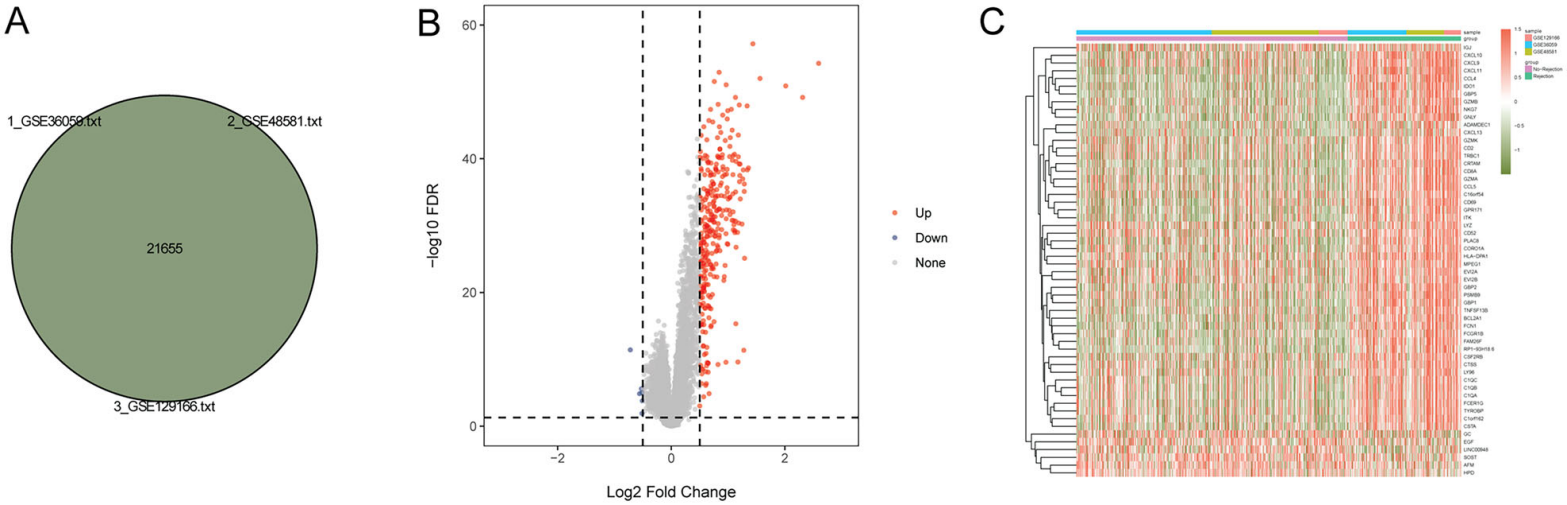
Identification of DEGs in the kidney transplant patients with allograft rejection. (A) Venn diagram of intersection genes in the training set; (B) volcano map of DEGs; (C) heat map of DEGs.

After data preprocessing, 21,655 intersection genes were identified in the training set (GSE36059, GSE48581, and GSE129166) (Figure 1(A)). A total of 319 DEGs were identified in the kidney transplant patients with allograft rejection, including 313 up-regulated and six down-regulated genes. Volcano map and heat map of all DEGs are shown in Figure 1(B,C), respectively.

### WGCNA

**Figure 2.**
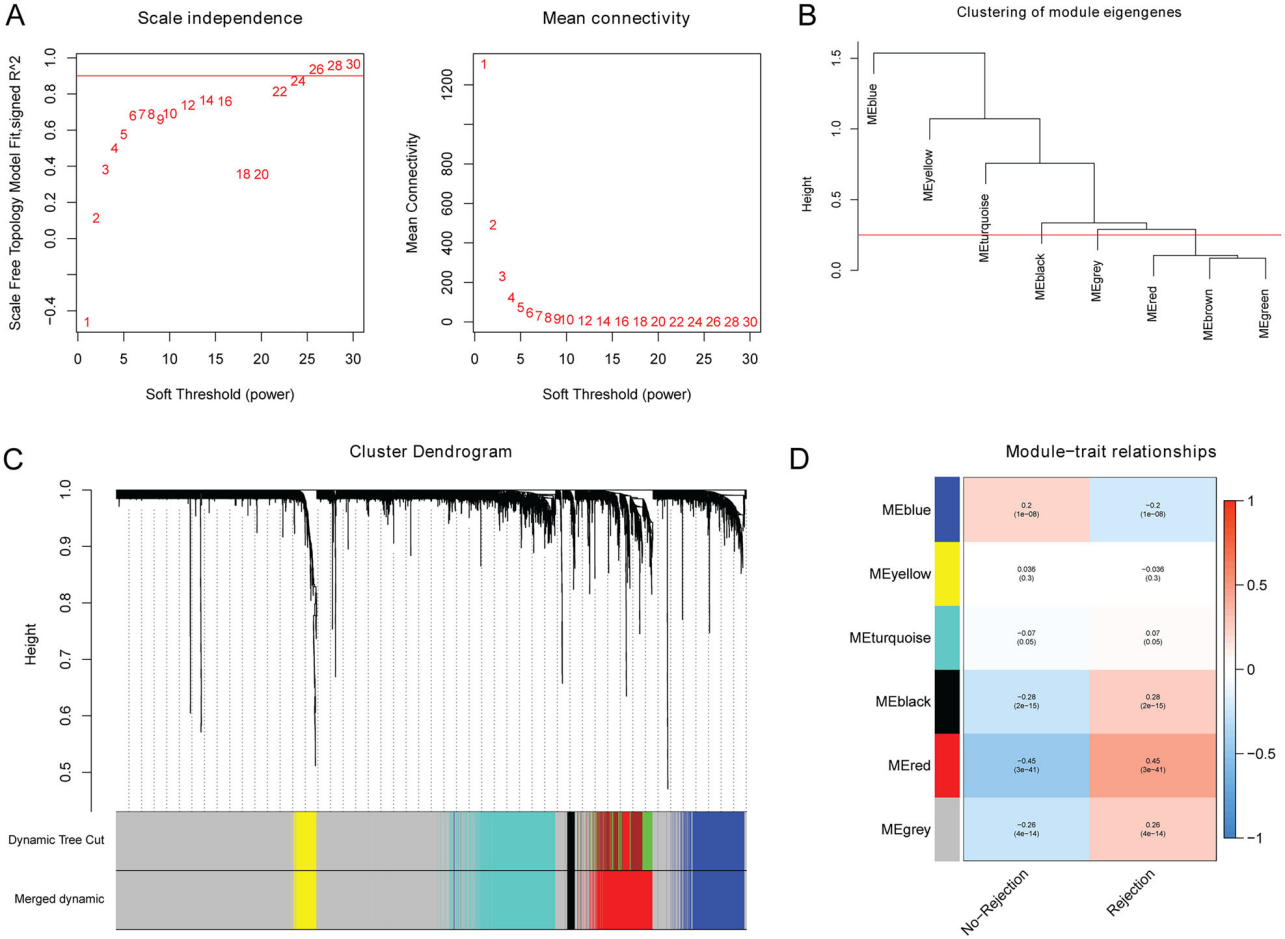
WGCNA in the kidney transplant patients with allograft rejection. (A) Scale-free fitting index of different soft threshold power and average connectivity of various soft threshold power; (B) merging of modules; (C) genes are divided into different modules; (D) correlation heat map between modular characteristic genes and allograft rejection.

WGCNA was used to identify genes related to allograft rejection after the kidney transplant. First, samples were clustered and four abnormal samples were deleted. When the parameter value of the weight coefficient is 24, the scale-free topology is approximate (Figure 2(A)). After building the cluster tree, the minimum number of genes in modules was set to 100, which separate seven modules (gray modules were not included). The dynamic cutting tree method was utilized to merge the modules with the dissimilarity degree <25%. Finally, five modules were identified (Figure 2(B,C)). As shown in Figure 2(D), the red module had the highest positive correlation with allograft rejection after kidney transplant (Pearson’s *r* = 0.45; *p* = 3E–41). Some up-regulated genes in the red module were identified, such as C-C motif chemokine receptor 5 (CCR5), CD86 molecule (CD86), CD8a molecule (CD8A), integrin subunit alpha M (ITGAM), and protein tyrosine phosphatase receptor type C (PTPRC). The blue module had the highest negative correlation with allograft rejection after kidney transplant (Pearson’s *r* = −0.2; *p* = 1E–08). Some down-regulated genes in the blue module were identified, such as 4-hydroxyphenylpyruvate dioxygenase (HPD) and afamin (AFM). Therefore, red and blue modules were chosen as hub modules, which involved 1066 genes.

### Functional analysis of common genes in hub modules and DEGs

**Figure 3.**
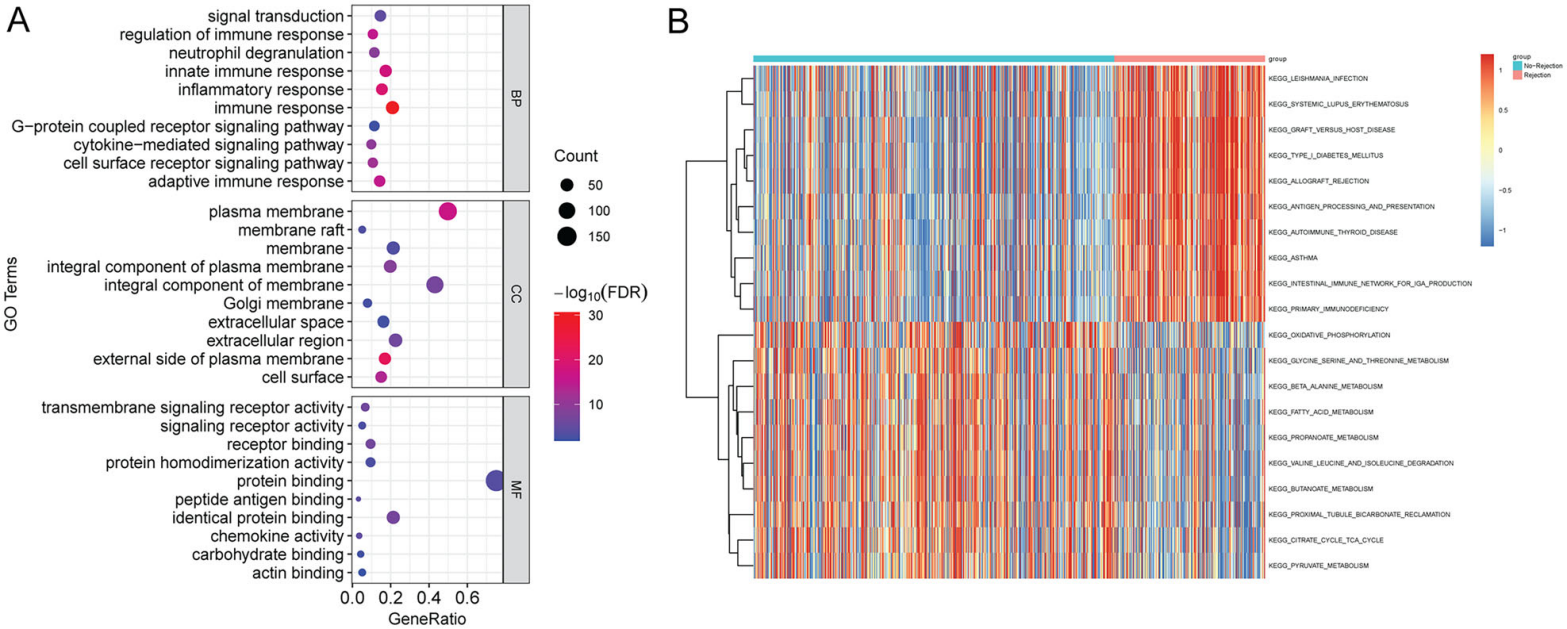
Functional analysis of common genes in hub modules and DEGs in the kidney transplant patients with allograft rejection. (A) GO analysis. BP: top 10 biological processes; CC: top 10 cytological components; MF: top 10 molecular functions; (B) top 10 metabolic pathways.

Totally, 270 common genes were identified in hub modules (involving 1066 genes) and DEGs (involving 319 genes) in the kidney transplant patients with allograft rejection. Based on GO analysis, immune response, external side of plasma membrane and identical protein binding were the most significantly enriched biological process, cytological component, and molecular function, respectively (Figure 3(A)). In the GSVA analysis, a total of 148 metabolic pathways were identified. Some metabolic pathways were more active in the allograft rejection group, such as graft versus host disease and type I diabetes mellitus (Figure 3(B)).

### PPI analysis of common genes in hub modules and DEGs

**Figure 4.**
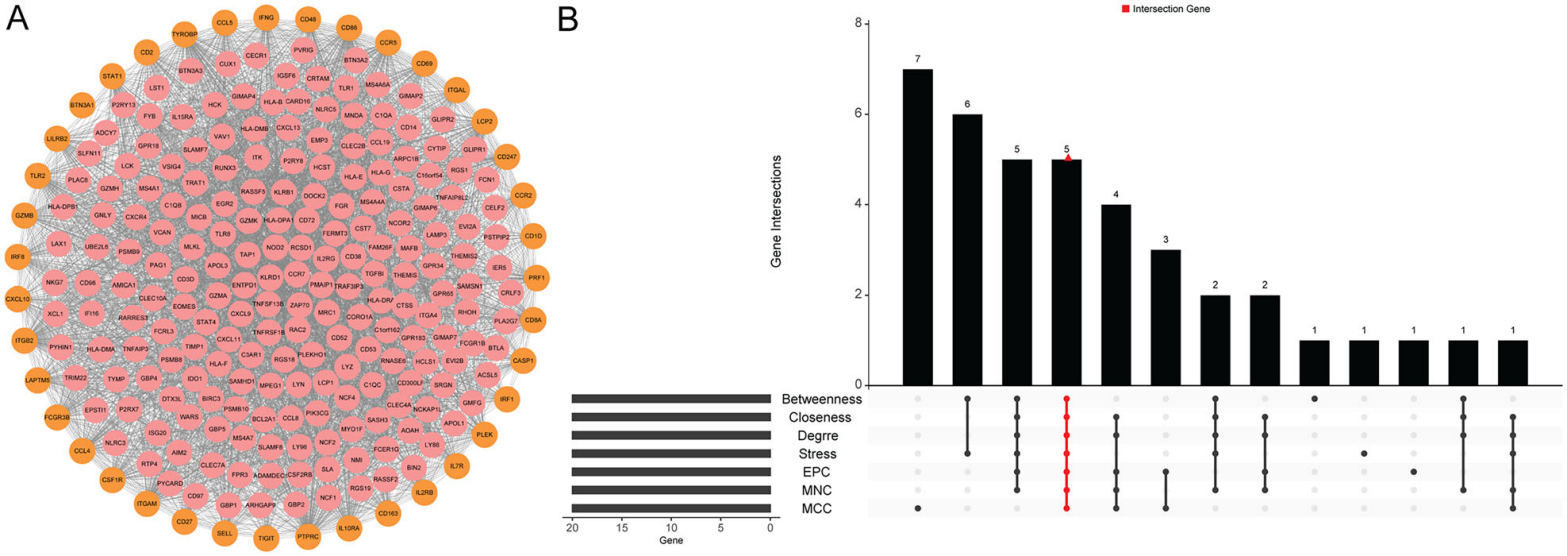
PPI analysis of common genes in hub modules and DEGs in the kidney transplant patients with allograft rejection. (A) PPI network constructed by common genes; (B) identification of key genes by intersecting the top 20 genes of 7 topology analysis methods.

**Figure 5.**
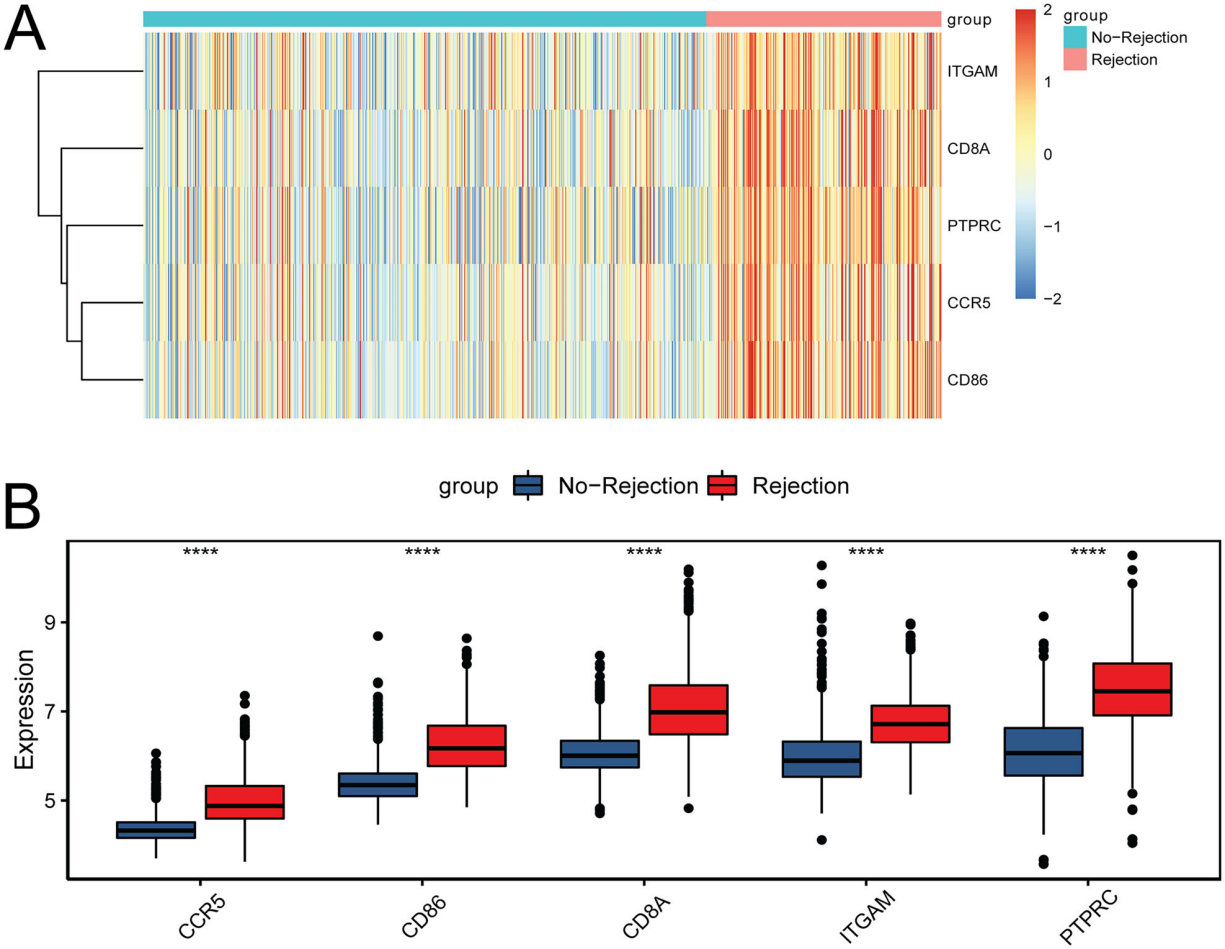
The heat map (A), expression validation (B) of 5 key genes in the kidney transplant patients with allograft rejection. ****p < 0.0001.

These 270 common genes were put into STRING database to study the regulatory relationship between proteins encoded by these genes in kidney transplant patients with allograft rejection (Figure 4(A)). Outermost 39 genes were derived from the union of the first 20 genes of seven topology analysis methods. After screening of the first 20 node genes of each algorithm score, a total of five key genes were identified (Figure 4(B)), including CCR5, CD86, CD8A, ITGAM, and PTPRC. The heat map of the above five key genes is shown in Figure 5(A). Moreover, the up-regulation of the above five key genes was verified in the validation set (Figure 5(B)).

### Construction of diagnostic model based on five key genes

**Figure 6.**
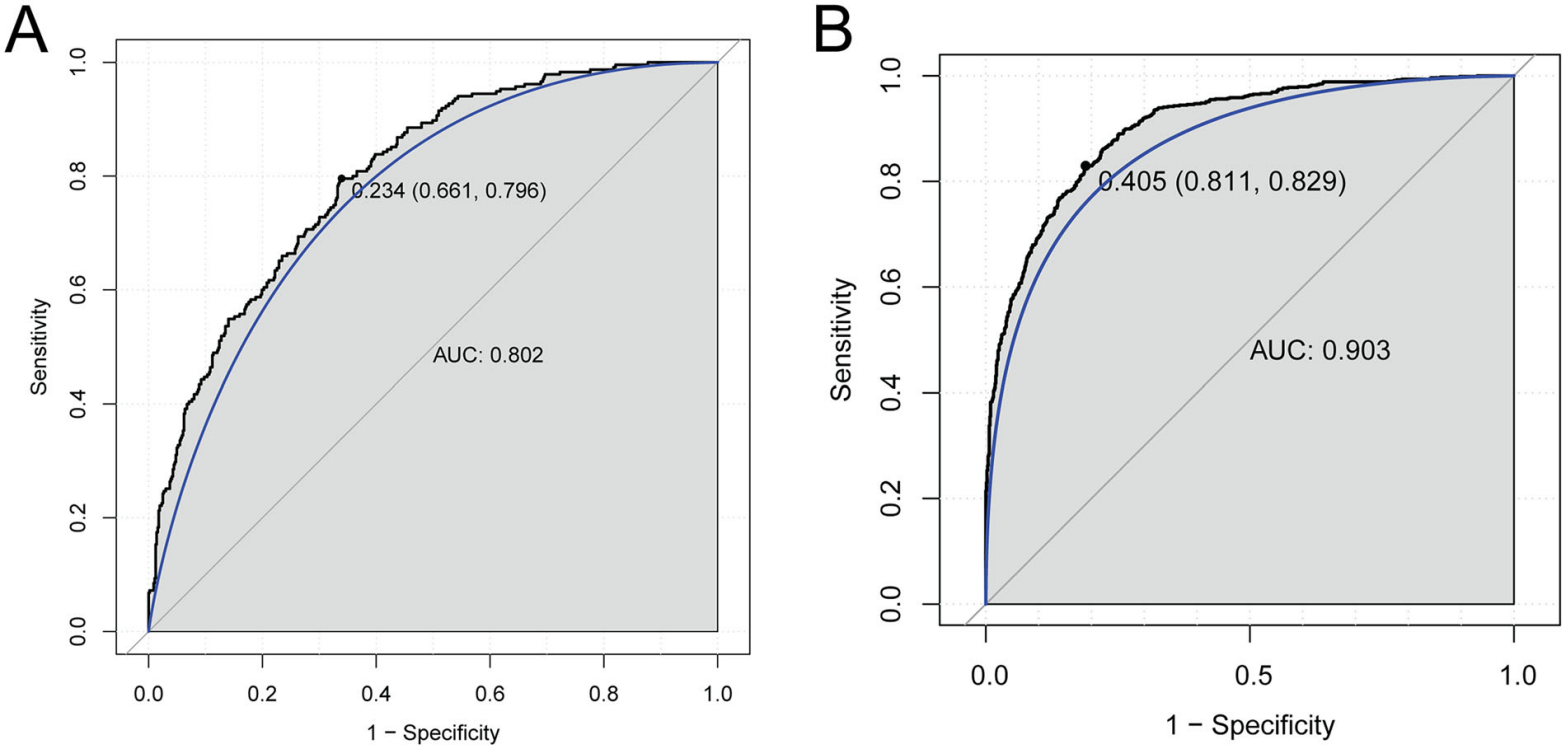
Construction of diagnostic model based on 5 key genes in the kidney transplant patients with allograft rejection. (A) ROC curve in the training set; (B) the ROC curve in the validation set.

**Figure 7.**
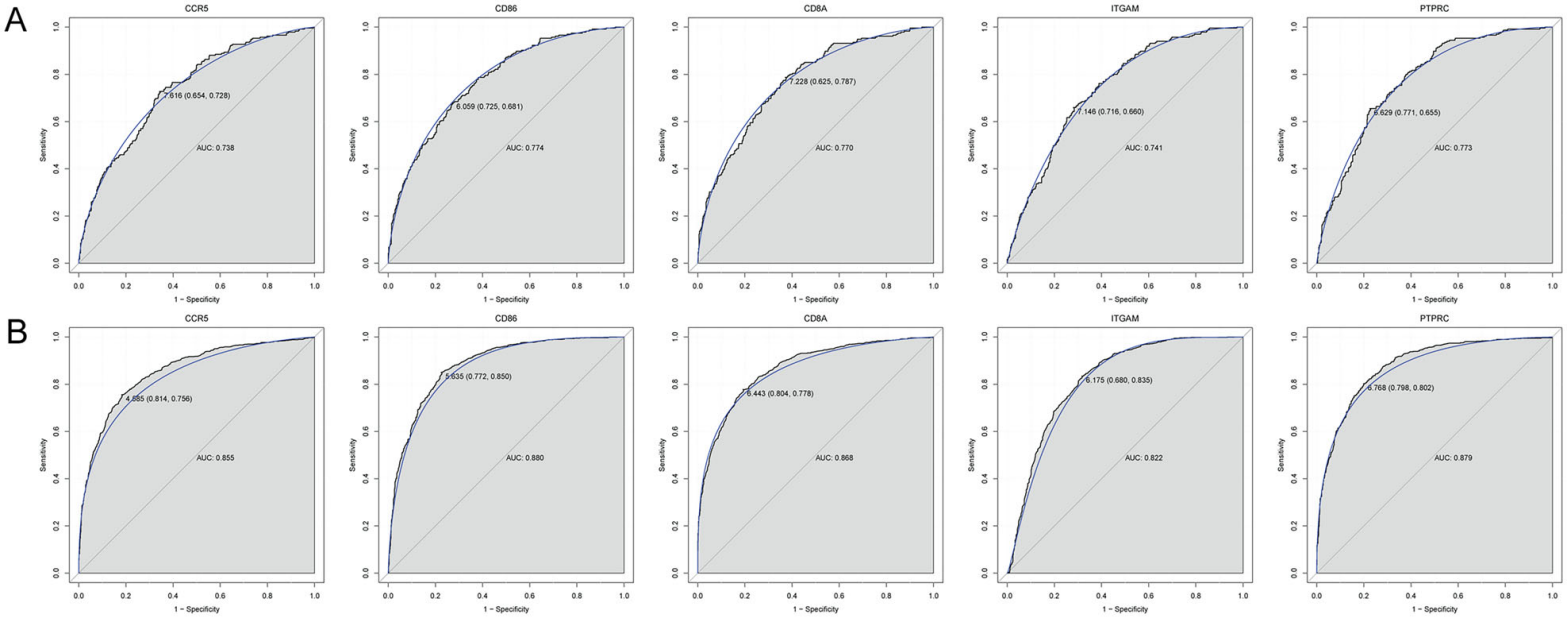
Diagnostic analysis of 5 single key genes in the training set (A) and validation set (B) in the kidney transplant patients with allograft rejection.

The diagnostic model for kidney transplant patients with allograft rejection was constructed based on five key genes in the training set (Figure 6(A)). AUC value was 0.802. The diagnostic model was also verified in the validation set (Figure 6(B)). The AUC value in the validation set was 0.903. This suggested that the diagnostic model based on five key genes had an excellent diagnostic performance for kidney transplant patients with allograft rejection. Additionally, the diagnostic value of the single key gene was analyzed in the training set (Figure 7(A)) and the validation set (Figure 7(B)). The AUC value of above five key genes was more than 0.7, which suggested a potential diagnostic value of these genes for kidney transplant patients with allograft rejection.

### Construction of regulatory networks between miRNAs, TFs, and five key genes

**Figure 8.**
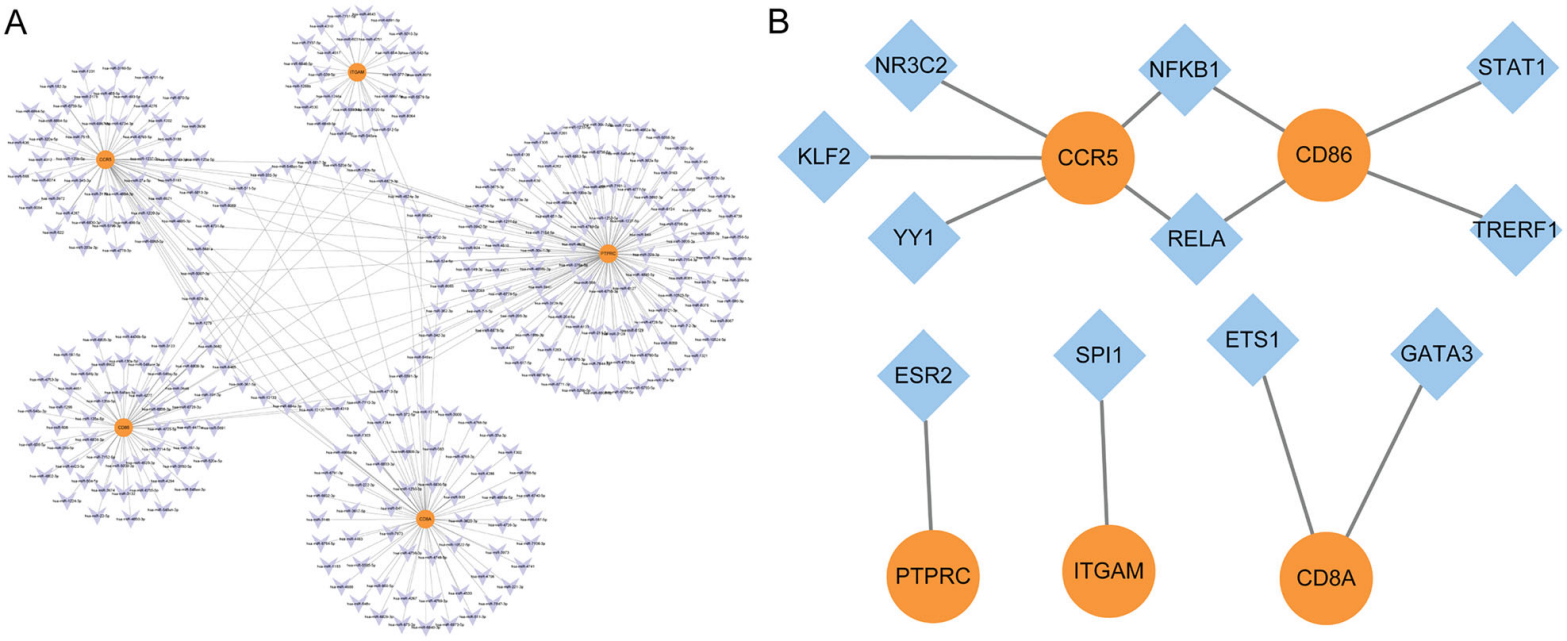
Construction of regulatory networks between miRNAs, TFs and 5 key genes in the kidney transplant patients with allograft rejection. (A) miRNA-key gene regulatory network. Purple and orange color represent miRNA and key gene, respectively; (B) TFs-key gene regulatory network. Orange and blue represent key gene and TF, respectively.

Based on interaction data of miRDB database, the miRNA-key gene regulatory network was constructed in kidney transplant patients with allograft rejection (Figure 8(A)). There were respectively 121, 30, 67, 60, and 65 related miRNA with PTPRC, ITGAM, CD8A, CD86, and CCR5. Three miRNA-key gene regulatory pairs were identified, including hsa-miR-8485-ITGAM/CD86, hsa-miR-12123-PTPRC, and hsa-miR-664a-3p-CCR5/CD8A. According to the TRRUST database, the role of TFs in regulation of five key genes was investigated (Figure 8(B)). It is noted that TFs of nuclear factor kappa B subunit 1 (NFKB1) and RELA proto-oncogene, NF-kB subunit (RELA) regulated the expression of CCR5 and CD86.

### Correlation analysis between immune and five key genes

**Figure 9.**
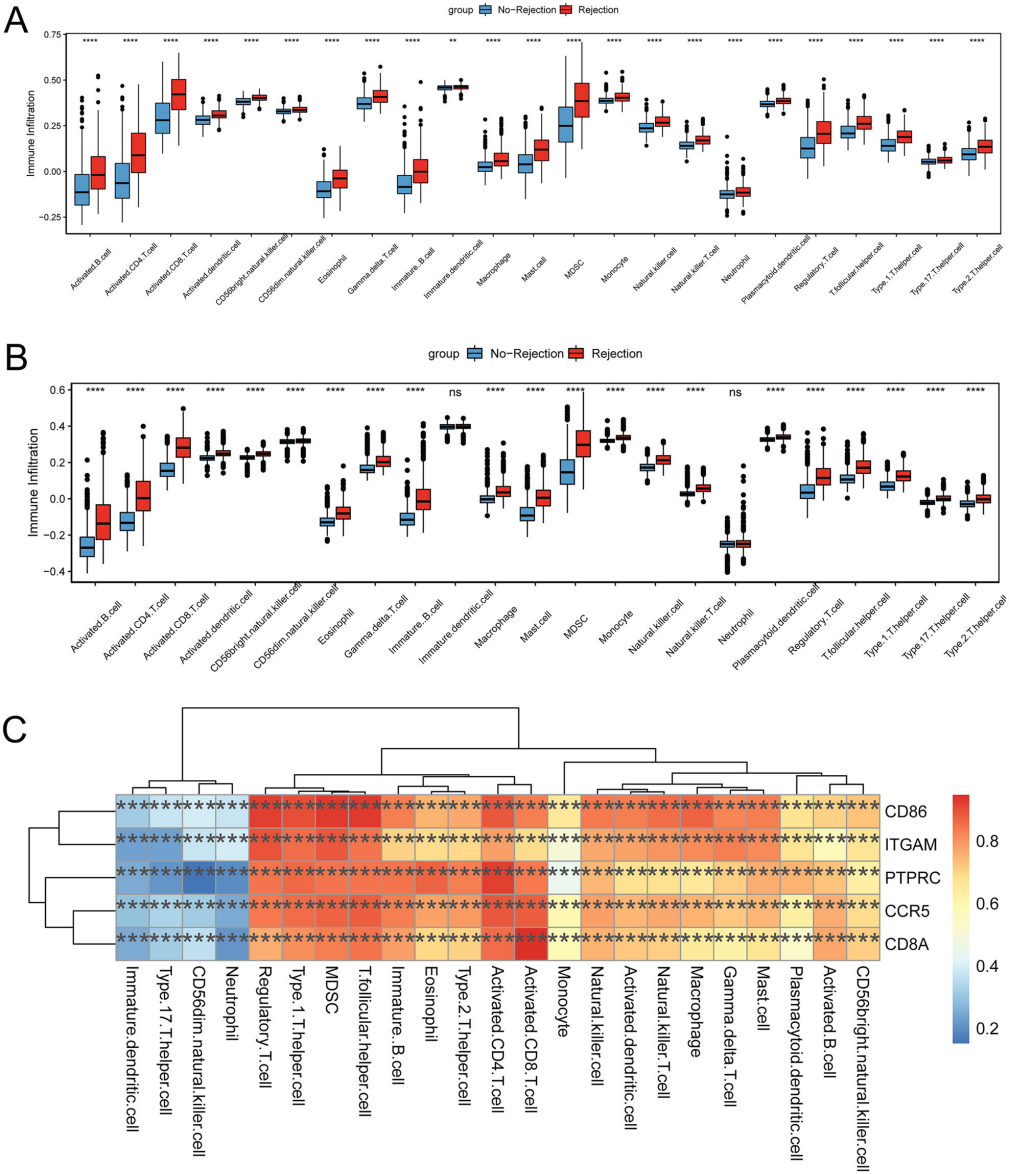
Correlation analysis between immune and 5 key genes in the kidney transplant patients with allograft rejection. (A) Differences in the degree of infiltration of 23 types of immune cells in the training set; (B) differences in the degree of infiltration of 23 types of immune cells in the validation set; (C) correlation heat map between 23 types of immune cells and 5 key genes. **p < 0.01, ***p < 0.001, ****p < 0.0001. ns: not significant.

The ssGSEA was used to evaluate the status of 23 types of immune cell infiltration in the training set in kidney transplant patients with allograft rejection (Figure 9(A)). Infiltration degree of 23 types of immune cells was high in the kidney transplant patients with allograft rejection. In the validation set (Figure 9(B)), apart from neutrophil and immature dendritic cells, the infiltration degree of the rest of 21 types of immune cells was elevated in kidney transplant patients with allograft rejection. Interestingly, all 23 types of immune cells were significantly positively correlated with five key genes (Figure 9(C)). For example, activated CD4 T cells, activated CD8 T cells, myeloid-derived suppressor cells (MDSCs), regulatory T cells, and T follicular helper cells were significantly positively correlated with PTPRC, CD8A, CD86, ITGAM, and CCR5, respectively.

### Correlation analysis between hypoxia and five key genes

**Figure 10.**
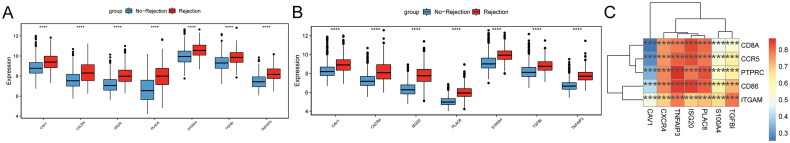
Correlation analysis between hypoxia and 5 key genes in the kidney transplant patients with allograft rejection. (A) Expression of 7 hypoxia-related genes in the training set; (B) expression of 7 hypoxia-related genes in the validation set; (C) correlation heat map between 7 hypoxia-related genes and 5 key genes. ***p < 0.001, ****p < 0.0001.

There are 200 hypoxia-related genes in the MSigDB database. Moreover, these 200 genes are highly expressed in hypoxia state. A total of seven common genes were identified between 200 hypoxia-related genes and 319 DEGs, including caveolin 1 (CAV1), C-X-C motif chemokine receptor 4 (CXCR4), interferon stimulated exonuclease gene 20 (ISG20), placenta associated 8 (PLAC8), S100 calcium binding protein A4 (S100A4), transforming growth factor beta induced (TGFBI), and TNF alpha induced protein 3 (TNFAIP3). These seven genes were up-regulated in kidney transplant patients with allograft rejection in training set (Figure 10(A)) and validation set (Figure 10(B)). Moreover, all seven hypoxia-related genes were significantly positively correlated with five key genes (Figure 10(C)). It is noted that TNFAIP3, ISG20, PLAC8, TGFBI, and CXCR4 were significantly positively correlated with PTPRC, CD8A, CD86, ITGAM, and CCR5, respectively.

### Correlation analysis between ferroptosis and five key genes

**Figure 11.**
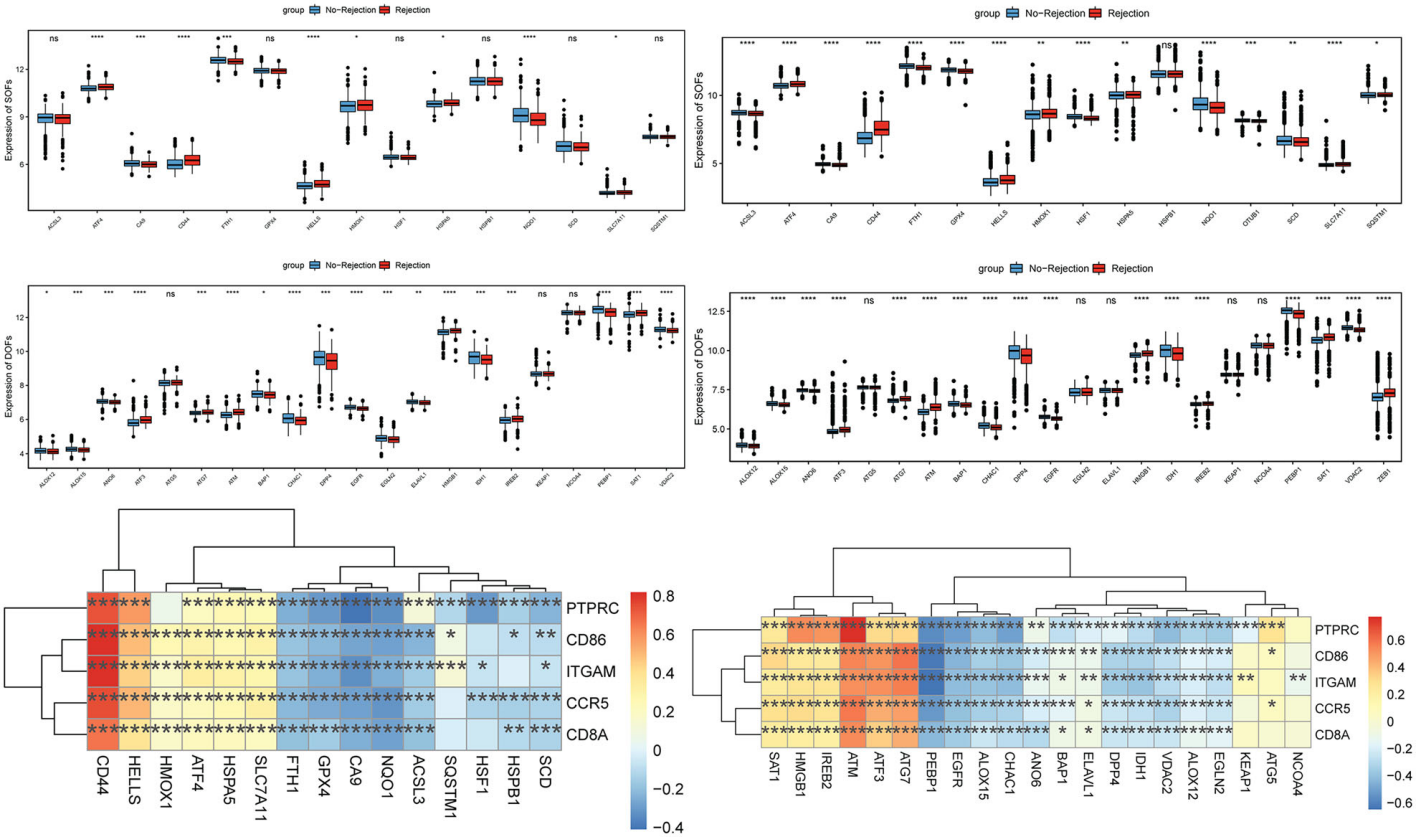
Correlation analysis between ferroptosis and 5 key genes in the kidney transplant patients with allograft rejection. (A) Expression of SOFs in training set; (B) expression of SOFs in validation set; (C) expression of DOFs in training set; (D) expression of DOFs in validation set; (E) correlation heat map between SOFs and 5 key genes; (F) correlation heat map between DOFs and 5 key genes. *p < 0.05, **p < 0.01, ***p < 0.001, ****p < 0.0001. ns: not significant.

Ferroptosis status was predicted based on the suppressors of ferroptosis (SOFs) and drivers of ferroptosis (DOFs) in the literature. Some SOFs, such as CD44 molecule (CD44) and carbonic anhydrase 9 (CA9) were respectively significantly up-regulated and down-regulated in kidney transplant patients with allograft rejection in training set (Figure 11(A)) and validation set (Figure 11(B)). Some DOFs, such as ATM serine/threonine kinase (ATM) and phosphatidylethanolamine binding protein 1 (PEBP1) were respectively significantly up-regulated and down-regulated in kidney transplant patients with allograft rejection in training set (Figure 11(C)) and validation set (Figure 11(D)). Depending on the correlation analysis between SOFs and five key genes (Figure 11(E)), CD44 was significantly positively correlated with five key genes. CA9 was the most significantly negatively correlated with PTPRC. According to the correlation analysis between DOFs and five key genes (Figure 11(F)), ATM was the most significantly positively correlated with PTPRC. PEBP1 was the most significantly positively negatively with ITGAM.

### Correlation analysis between EMT and five key genes

**Figure 12.**
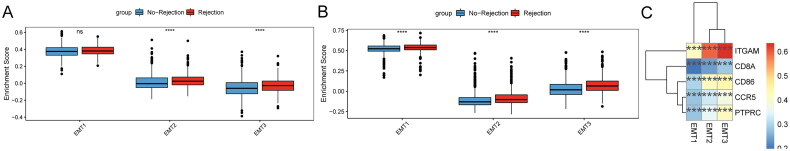
Correlation analysis between EMT and 5 key genes in the kidney transplant patients with allograft rejection. (A) Status evaluation of EMT in the training set; (B) status evaluation of EMT in the validation set; (C) correlation heat map between EMT and 5 key genes. ***p < 0.001, ****p < 0.0001. ns: not significant.

Based on the evaluation of EMT status, EMT2 and EMT3 were higher in the kidney transplant patients with allograft rejection (Figure 12(A)). Similarly, EMT2 and EMT3 were higher in the kidney transplant patients with allograft rejection in the validation set (Figure 12(B)). It is worth mentioning that EMT2 and EMT3 were significantly positively correlated with ITGAM (Figure 12(C)).

### Drug prediction of five key genes

**Figure 13.**
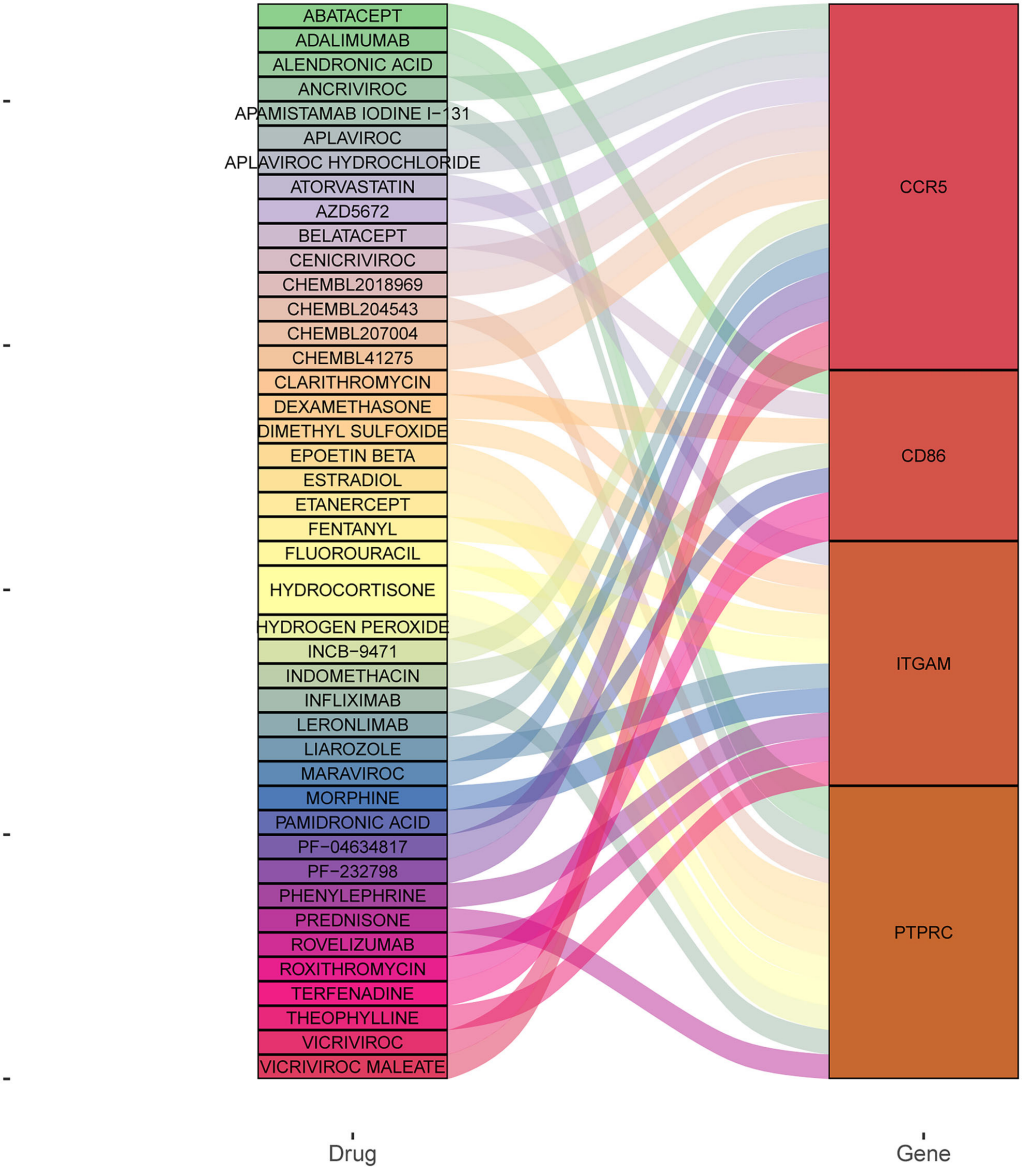
Drug prediction of 4 key genes in the kidney transplant patients with allograft rejection.

Drugs associated with four key genes were screened based on DGIdb database (Figure 13). It is a pity that no related drugs were found for CD8A in the DGIdb database. CD86, CCR5, and ITGAM were respectively drug target of ABATACEPT, MARAVIROC, and CLARITHROMYCIN. In addition, PTPRC was drug target of both PREDNISONE and EPOETIN BETA.

## Discussion

CCR5, a chemokine receptor, is associated with the pathogenesis of a wide spectrum of health conditions, such as inflammatory diseases and autoimmune diseases. In a rat renal acute rejection model, CCR5 is significant up-regulated after allogeneic transplantation [28]. Interruption of the CCR5 is related to prolongation of allograft survival [29,30]. In addition, in kidney transplant recipients, those who are homozygous for CCR5 delta 32 have improved survival [31]. CD86, expressed on antigen-presenting cells, suppresses host immunity [32,33]. The numbers of circulating CD86^+^ after kidney transplant are significantly higher than those at pre-transplantation [34]. CD8A is significant up-regulated after kidney transplantation [28]. ITGAM, a member of the β2 integrin family of adhesion molecules, is expressed by cells of the myeloid lineage [35]. ITGAM is expressed by some kidney tubules. ITGAM plays essential roles in the adhesion of monocytes, macrophages, and the uptake of pathogens [36,37]. PTPRC is involved in regulating B cell and T cell receptor signaling. PTPRC is up-regulated in stable and acute kidney transplant patients [38,39]. In this study, CCR5, CD86, CD8A, ITGAM, and PTPRC were up-regulated and had the positive correlation with allograft rejection in kidney transplant patients. It is noted that a combination or single gene of the above five genes had a potential diagnostic value for kidney transplant patients with allograft rejection. Thus it can be seen that CCR5, CD86, CD8A, ITGAM, and PTPRC play crucial roles in the process of allograft rejection and can be considered as potential diagnostic markers for allograft rejection after the kidney transplant.

Both innate and adaptive immune systems play critical roles in allograft rejection after the kidney transplant, among which T lymphocytes are the main cells for recognizing allografts [40]. According to function, T cells are divided into CD4^+^ T cells, CD8^+^ T cells and Treg cells [41,42]. Significantly higher RNA expression levels of CD4 are found in blood samples of patients with T-cell-mediated kidney transplant rejection [43]. Natural killer (NK) cells interact directly with CD4^+^ T lymphocytes and induce acute rejection mechanisms [44]. CD8^+^ T lymphocytes infiltrate the kidney during allograft rejection [45]. CD8^+^ senescent T cells are linked to a reduced possibility of allograft rejection after kidney transplantation [46,47]. Relatively few effector memory CD8^+^ T cells and effector CD8^+^ T cells are found in the peripheral blood of patients receiving immunosuppressive therapy after kidney transplantation [48]. In peripheral blood of kidney transplant patients, low regulatory T cells are related to allograft rejection and poor outcomes [49–55]. In addition, regulatory T cells can suppress memory CD8^+^ T cell and contribute to allograft survival [56]. T follicular helper cells induce differentiation of B lymphocyte and contribute to rejection [57–60]. Inhibition differentiation and function of T follicular helper cell can prevent the development of anti-donor antibody responses in transplantation [61–63]. In kidney transplantation, MDSCs reveal a strong immune suppressive ability [64]. In kidney transplant patients, MDSCs expand T regulatory cells [65]. In the present study, all 23 types of immune cells were significantly positively correlated with CCR5, CD86, CD8A, ITGAM, and PTPRC. Moreover, activated CD4 T cells, activated CD8 T cells, MDSCs, regulatory T cells, and T follicular helper cells were significantly positively correlated with PTPRC, CD8A, CD86, ITGAM, and CCR5, respectively. It is indicated that PTPRC, CD8A, CD86, ITGAM, and CCR5 may play key roles in the immune systems, which are associated with allograft rejection after the kidney transplant.

In allografts, local over expression of vascular endothelial growth factor (VEGF) results in chronic rejection. Hypoxia is the major stimulating factor of VEGF expression [66,67]. Herein, seven hypoxia-related genes were up-regulated in kidney transplant patients with allograft rejection. It is noted that TNFAIP3, ISG20, PLAC8, TGFBI, and CXCR4 were significantly positively correlated with PTPRC, CD8A, CD86, ITGAM, and CCR5, respectively. In kidney transplantation, TNFAIP3 expression is linked to outcome prediction [68]. ISG20 is up-regulated in acute rejection after kidney transplant [69]. ISG20 could be a novel therapeutic target of renal fibrosis [70]. TGFBI can promote renal fibrosis [71]. The antagonist of CXCR4 effectively reduces the rejection intensity after transplantation [72,73]. The positive correlation between hypoxia-related genes and PTPRC, CD8A, CD86, ITGAM, and CCR5 may be associated with allograft rejection after the kidney transplant.

Ferroptosis is considered to play key regulatory roles in acute kidney injury. However, the role of ferroptosis in immune rejection after kidney transplantation remains unclear [74]. In this study, two SOFs, CD44, and CA9 were respectively significantly up-regulated and down-regulated in kidney transplant patients with allograft rejection. Two DOFs, ATM, and PEBP1 were respectively significantly up-regulated and down-regulated in kidney transplant patients with allograft rejection. There is a prominent continuous expression of CD44 by the endothelial cells of kidney allograft in acute rejection [75]. CD44 absence leads to attenuated kidney injury following ischemia or reperfusion injury [76]. CA9, a membrane protein, regulates cell proliferation in response to hypoxia [77,78]. CA9 can serve as a potential target for renal cell carcinoma-specific immunotherapy [79]. Activation of ATM is found in renal ischemia or reperfusion injury [80]. PEBP1, plays roles in anti-inflammatory effects under homeostatic/basal conditions, is associated with kidney allograft rejection [81,82]. This suggested the association of ferroptosis and allograft rejection after kidney transplantation. In addition, CD44 was significantly positively correlated with PTPRC, CD8A, CD86, ITGAM, and CCR5. CA9 and ATM were respectively the most significantly negatively and positively correlated with PTPRC. PEBP1 was the most significantly positively negatively with ITGAM. These results indicated that SOFs (CD44 and CA9) and DOFs (ATM and PEBP1) showed a complex regulatory relationship between ferroptosis and PTPRC, CD8A, CD86, ITGAM, and CCR5.

EMT plays key roles in the fibrosis process of renal grafts [83]. In the present study, EMT2 and EMT3 were higher in the kidney transplant patients with allograft rejection. Moreover, EMT2 and EMT3 were significantly positively associated with ITGAM. EMT2 and EMT3 were significantly linked to renal cell carcinoma [84]. Positively correlation between EMT2, EMT3, and ITGAM may be involved in the fibrosis process after kidney transplant.

Based on regulatory networks between miRNAs and PTPRC, CD8A, CD86, ITGAM, and CCR5, three miRNA-key gene regulatory pairs were identified, including hsa-miR-8485-ITGAM/CD86, hsa-miR-12123-PTPRC, and hsa-miR-664a-3p-CCR5/CD8A. In addition, TFs of NFKB1 and RELA regulated the expression of CCR5 and CD86. NFKB1 is an inflammatory marker. After kidney transplantation, the NFKB1 promoter polymorphism (-94ins/delATTG) is related to susceptibility to cytomegalovirus infection [85]. Increased expression of RELA is associated with renal thrombotic microangiopathy [86]. Our result suggested that the regulation relationship between miRNA, TFs and PTPRC, CD8A, CD86, ITGAM, and CCR5 could be associated with inflammatory response in the development of allograft rejection after the kidney transplant.

It is reported that existing immunosuppressive drugs are not sufficient to completely prevent allograft rejection in kidney transplant patients [87,88]. Therefore, it is needed to find potential drug targets for kidney transplant patients with allograft rejection. Based on DGIdb database, PTPRC was drug target of both PREDNISONE and EPOETIN BETA. In addition, CD86, CCR5, and ITGAM were respectively drug target of ABATACEPT, MARAVIROC, and CLARITHROMYCIN. PREDNISONE is an essential component of immunosuppression protocols during the first three decades of clinical kidney transplantation [89]. Anemia is a common complication of kidney transplantation. In kidney transplant recipients with moderate renal insufficiency, correction of anemia with EPOETIN BETA can slow the decline in glomerular filtration rate, reduce the incidence of end-stage renal disease, and improve quality of life without increasing the risk of cardiovascular events [90].

Treatment success of ABATACEPT has been found in post-kidney transplant patients [91]. MARAVIROC impairs lymphocyte chemotaxis with a theoretical reduction in organ transplant rejection [92]. CLARITHROMYCIN is utilized to prevent and treat infection in kidney transplant recipients [93]. Thus, it can be seen that PTPRC, CD86, CCR5, and ITGAM could be considered as potential targets of PREDNISONE, EPOETIN BETA, ABATACEPT, MARAVIROC, and CLARITHROMYCIN, which may provide novel treatment options for kidney transplant patients with allograft rejection.

Beside above five diagnostic key genes positively correlated with allograft rejection, two genes negatively correlated with allograft rejection were found, including HPD and AFM. HPD is down-regulated in renal ischemia–reperfusion injury [94]. AFM is a biomarker of acute kidney transplant rejection [95]. Decrement in AFM is observed in early acute kidney allograft rejection [96]. It is reported that decreased expression of HPD and AFM may be associated with allograft rejection after the kidney transplant. In addition, based on GSVA analysis, some metabolic pathways were more active in the allograft rejection group, such as graft versus host disease and type I diabetes mellitus. Graft versus host disease is a rare complication after kidney transplantation [97]. New-onset diabetes after transplantation, another complication in kidney transplant recipients, can increase the risk of infections, allograft loss, and mortality [98,99].

In conclusion, five diagnostic genes were identified in kidney transplantation patients with allograft rejection, including CCR5, CD86, CD8A, ITGAM, and PTPRC. Highly infiltrated immune cells, highly expression of hypoxia-related genes and activated status of EMT were significantly positively related to these diagnostic genes. SOFs and DOFs showed a complex regulatory relationship between ferroptosis and five diagnostic genes. CD86, CCR5, and ITGAM were respectively drug target of ABATACEPT, MARAVIROC, and CLARITHROMYCIN. PTPRC was drug target of both PREDNISONE and EPOETIN BETA. Our study could be useful in understanding changes in the microenvironment within kidney transplantation. However, there are limitations to our study. First, the mRNA or protein expression validation analysis of CCR5, CD86, CD8A, ITGAM, and PTPRC is needed in kidney transplant biopsy sample from transplant recipients with graft rejection compared to who do not present dysfunction events. Second, the potential pathological mechanism of these genes should be investigated in cell lines or animal models. Third, the potential interaction mechanism between immune cell and CCR5, CD86, CD8A, ITGAM, and PTPRC are needed to investigate in the future study.

## Data Availability

The data are available in the manuscript.
